# The effects of sub-chronic administration of sub-lethal doses of amitraz/xylene on selected reproductive parameters of male Wistar rats

**Published:** 2016

**Authors:** V. U. Omoja, S. M. Anika, I. U. Asuzu

**Affiliations:** 1 Department of Veterinary Physiology and Pharmacology, Faculty of Veterinary Medicine, University of Nigeria, Nsukka Enugu State, Nigeria;; 2 Office of the Vice-Chancellor, Federal University Oye-Ekiti, Ekiti State, Nigeria

**Keywords:** Amitraz, Rat, Sperm, Testis, Testosterone

## Abstract

This study investigated the effects of sub-chronic administration of sub-lethal doses of amitraz on some testicular parameters of Albino rats. Twenty-four adult male Albino rats (100 ± 10 g) randomly assigned into four groups were used for the study. Groups A, B and C received 10.0, 2.0 and 0.4 mg/kg amitraz in 10 ml/kg water while group D received equivalent volume of water orally and daily for 84 days. Serum testosterone levels (TESL) were assessed on days 0, 28, 56 and 84. Epididymal sperm reserve (ESR), testicular sperm reserve (TSR), testicular weight index (TWI) and testicular histology were evaluated at the end of the experiment. Results revealed dose-dependent reduction (P<0.05) in the mean TESL, ESR and TSR in the amitraz-treated groups as the dose of the amitraz increased. Histological study revealed testicular degeneration characterized by depopulation of seminiferous tubules and depletion of the spermatogenic cells in rats in group A. It was concluded that sub-chronic administration of sub-lethal doses of amitraz could lead to reduced sperm quantity.

## Introduction

Amitraz is a formamidine pesticide marketed world-wide as an acaricide and insecticide since 1974 (Crofton et al., 1989 ▶). It is labeled for topical use to control ticks, mites and lice on cattle, pigs, poultry and dogs. Commercial formulations of amitraz incorporate organic solvents such as xylene to the active agent in order to stabilize the agent and potentiate its ability to eradicate pests. Therefore, when humans and animals are exposed to the pesticides, the symptoms and signs of toxicity observed may partly be those of the organic solvent like xylene and partly to the agent (Gwaltney-Brant, 2004 ▶). Ectoparasite resistance to amitraz could increase human, animal and plant exposure to the toxicological effects of pesticide by giving higher doses of the agent in order to achieve the desired effect (Soberans et al., 2002 ▶). There is information on the toxicological effects of amitraz on the central nervous system including its effects on the eye, cardiovascular, respiratory, gastrointestinal, hyper-glycemic as well as its anti-inflammatory effects (Hsu and Schaffer, 1988 ▶). Amitraz has been shown to induce cytochrome P450-dependent monooxygenases in the liver of treated rats (Ueng et al., 2004 ▶). Free oxygen radicals are produced during amitraz oxidation (Kruk and Bounias, 1992 ▶), indicating the potential for cytotoxicity (Bukowska, 2003 ▶). Sub-chronic administration of sub-lethal doses of amitraz to rats led to liver and kidney damage (Omoja et al., 2015 ▶). However, limited information is available on the effects of amitraz on the testicular weight index (TWI), sperm reserve and testicular histology of amitraz-exposed animals. This research is therefore designed to study the effects of sub-chronic administration of sub-lethal doses of amitraz on these testicular parameters of amitraz-exposed Albino Wistar rats.

## Materials and Methods

The amitraz used was Zamitraz^®^ a product of Zampharm Ltd. London, England containing 12.5% solution of amitraz (125 mg/ml) with batch number 120406; manufacturing date of 12/04/2012 and expiration date of 11/04/2015. Live animal procedures used in this study were approved by the University Institutional Animal Care and Use Committee that equally granted the permission to use the animals in this toxicity study. The Albino Wistar rats were obtained from a stock bred and maintained at the Department of Veterinary Physiology and Pharmacology, University of Nigeria, Nsukka. The rats were kept in aluminum cages, allowed two weeks of acclimatization and fed standard commercial rat pellets. Portable water was allowed *ad libitum*. Four groups of male rats weighing 100 ± 10 g were used in this study and each group was made up of six rats. The rats were treated daily as follows: 10.0, 2.0, and 0.2 mg/kg amitraz in 10 ml/kg water for group A, B and C, respectively, while group D given equivalent water, served as the control. The doses used were derived from the acute toxicity study of amitraz in rats (Omoja, 2015 ▶). Blood samples were collected through the median canthus of rats and allowed to clot and centrifuged at 2000 rpm for 15 min. The sera were aspirated into properly labelled vials and stored at -20°C until required for testosterone assay. Serum testosterone levels (TESL) were assayed using Testosterone AccuBind^TM^ Microplate Enzyme Immunoassay Test Kit (Monobind Inc., Lake Forest, USA). The epididymides (right and left sides) and the testes (right and left sides) were dissected out and weighed in a sensitive balance before being transferred into individual clean test tubes, and minced with ophthalmologic scissors and homogenized for 1 min in 20 ml of phosphate-buffered saline (Oishi, 2002 ▶) for determination of epidydimal sperm reserve (ESR) and testicular sperm reserve (TSR), respectively. The homogenate was later filtered using a nylon mesh sieve and 20 μL aliquots of the filtered homogenate fluid were used in charging the Neubaur chamber for the appropriate counting of the number of sperms × 10^6^ per ml of tissue sample. Tissue sections of the testis from amitraz-exposed and control rats were fixed in 10% formol saline and dehydrated in ascending grades of ethanol. Thereafter, the tissues were cleared in chloroform overnight, infiltrated and embedded in molten paraffin wax. The blocks were later trimmed and sectioned at 5-6 μ. The sections were deparaffinized in xylene, taken to water and subsequently stained with haematoxylin and eosin (H&E) for light microscopy (Bancroft and Stevens, 1977 ▶). The data collected from this study were summarized as means with standard errors of the means and comparison of means was done by one-way ANOVA with least significant difference at the probability of 5% (Chatfield, 1983 ▶).

## Results

**Fig. 1:**
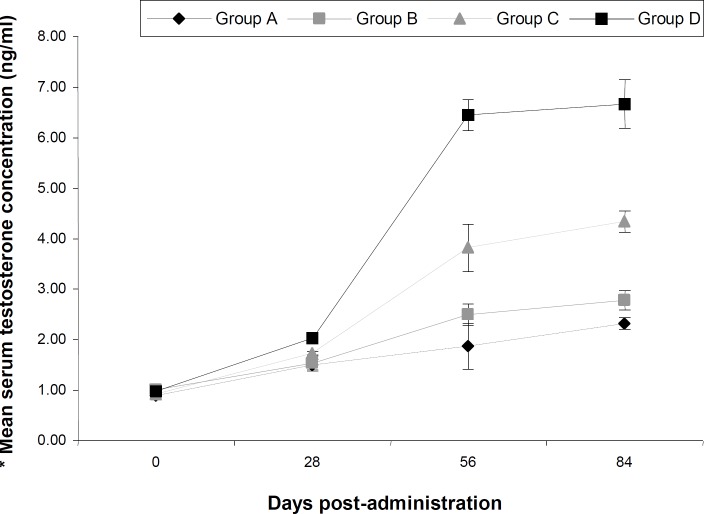
Mean serum testosterone levels of rats given oral sub-chronic amitraz treatment. Groups A, B, and C treated with amitraz at the dosages of 10.0, 2.0, and 0.4 mg/kg body weight, respectively; group D was treated with water at the dosage of 10.0 ml/kg. Mean±SEM based on 4 observations

**Fig. 2:**
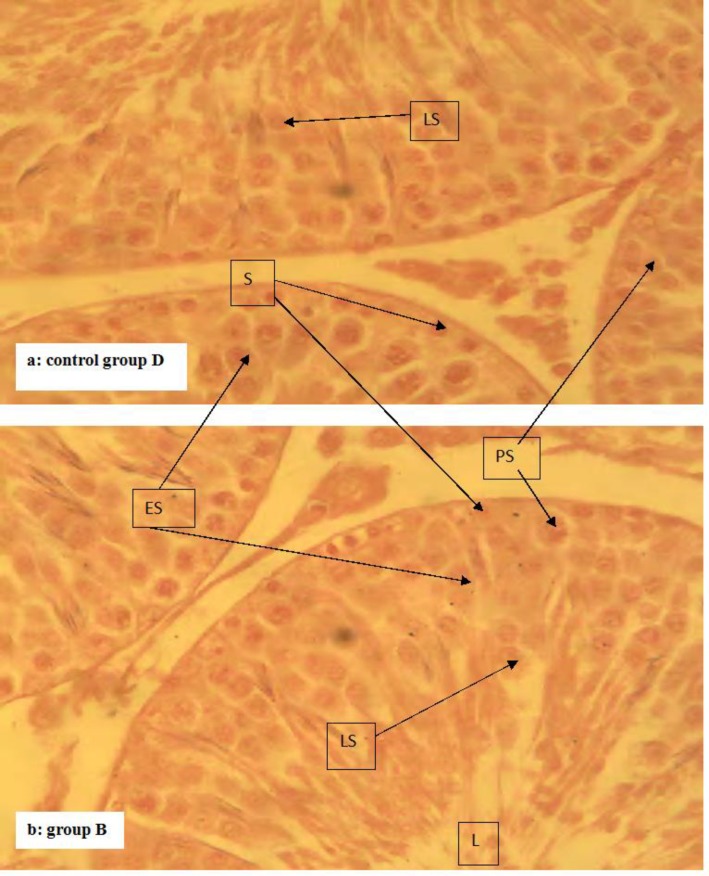
The histology of groups D (Fig. 2a, control group) and B: Note that the testicular histology of rats in this group B treated with 2.0 mg/kg amitraz (Fig. 2b) is similar to those of group C treated with 0.2 mg/kg amitraz (not shown) as well as the control group D given 10 ml/kg water (Fig. 2a). They show normal testicular histo-architecture. The symbols LS, ES, PS, S and L represent late spermatids, early spermatids, primary spermatids, spermatogonium and tubular lumen, respectively (H&E, ×400

**Fig. 3:**
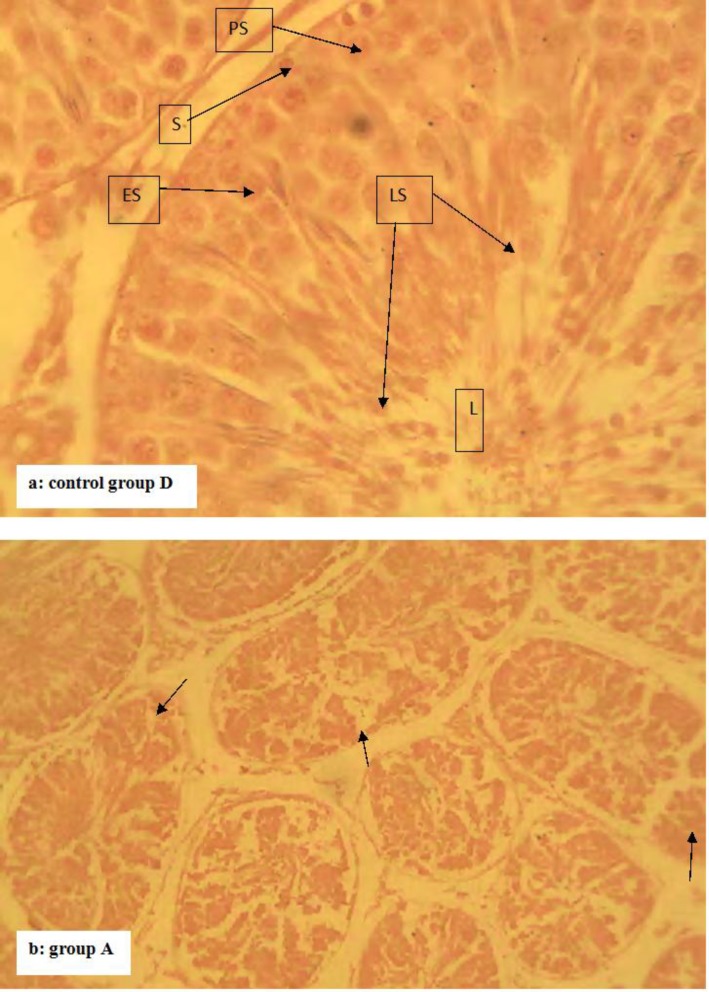
The histology of groups D (control group) and A: The control group D given 10 ml/kg water (Fig. 3a) show normal testicular histo-architecture. The symbols LS, ES, PS, S and L represent late spermatids, early spermatids, primary spermatids, spermatogonium and tubular lumen, respectively (H&E, ×400). The testicular histology of the section from rat in group A (Fig. 3b) treated with 10 mg/kg amitraz daily for 84 days using the oral route showed a diffuse degeneration of the seminiferous tubules (arrows) (H&E, ×100

The results of the body weight index (BWI) and testicular parameters (TSR, ESR, and TWI) of Albino Wistar rats given sub-chronic oral amitraz administration are presented in Table 1. The results revealed reduced (P<0.05) mean BWI of rats in group A when compared to groups B, C and D. There was no difference (P<0.05) between the mean BWI of rats in groups B, C and D. The mean ESR and TSR of rats in groups A and B were lower (P<0.05) than that of group C. There was no difference (P<0.05) between the mean ESR and TSR of rats in group A and B. The mean ESR and TSR of rats in group C was lower (P<0.05) than that of group D. The mean testosterone levels of rats did not vary (P<0.05) among the groups at day zero of the experiment. There was however a dose-dependent decrease among the groups as dosing continued from day 28 to the end of the experiment. Rats in groups A and B had reduced (P<0.05) mean testosterone levels when compared to group C from day 56 to the end of the experiment. The mean testosterone levels of rats in group C were lower (P<0.05) than that of group D between day 56 and end of the experiment (Fig. 1). Sections of the testis from rats in groups B, C and D showed no deviation from the normal testicular histo-architecture. It showed normal semi-niferous tubules lined by stratified epithelium of spermatogenic cells and sertoli cells (S). In between the basement membranes of the seminiferous tubules are thin vacular loose connective tissues which hold the leydig cells (Figs. 2a and b). However, sections of the testes from rats in group A showed a diffuse degeneration of the seminiferous tubules, characterized by depopulation of seminiferous tubules and depletion of the spermatogenic cells (Figs. 3a and b are a section of the testis from rat in group D showing normal testicular histo-architecture used to compare that from rat in group A (Fig. 3b)).

**Table 1:** The testicular parameters and body weight index of Albino Wister rats given sub-chronic amitraz treatment orally daily

Mean parameters (±SEM)	Treatment groups			
	A	B	C	D
Body weight index (%)	26.23 ± 7.31^a^	56.43 ± 6.06^b^	57.22 ± 6.18^b^	82.38 ± 12.64^b^
Epididymal sperm reserve (×10^6^/ml)	92.13 ± 1.34^a^	112.40 ± 3.76^a^	200.81 ± 7.30^b^	271.69 ± 14.97^c^
Testicular sperm reserve (×10^6^/ml)	102.00 ± 4.02^a^	126.51 ± 6.36^a^	269.25 ± 26.59^b^	448.11 ± 33.59^c^
Testicular weight index (%)	1.14 ± 0.05^a^	1.15 ± 0.03^a^	1.28 ± 0.02^b^	1.33 ± 0.03^b^

a, b, c Different superscripts represent significant difference at P≤0.05. Groups A, B, and C were treated with amitraz at the dosages of 10.0, 2.0, and 0.4 mg/kg body weight, respectively; group D was treated with water at the dosage of 10.0 ml/kg. SEM: Standard error of the mean

## Discussion

The BWI of rats in group A was lower than other amitraz-treated groups and the control group in this study suggesting that amitraz could reduce weight gain probably through reduction in feed consumption and oxidation of triglycerides. Farmers that use amitraz in the control of pests should weigh its advantage versus the weight loss associated with the continuous and high dosage usage, especially when applying amitraz during fattening. There was increase in testosterone level of all the rats at day 28 of the experiment, a finding which could be attributed to maturity of the rats. The reduction in testosterone levels observed with increase in the dose of amitraz suggests that increased dose of amitraz could possess the potential of reducing the libido of male animals. The importance of androgen on sexual behavior is well established (Shabsigh, 2005 ▶), and the proerectile effect of testosterone, the main androgen, is related to its interaction with the nitrergic system (Mills et al., 2004 ▶). Alternative treatment may be to combine other acaricides with low concentration of amitraz to achieve better eradication of pests in the animal. This combination therapy may solve the problem of resistance and toxicity as the drugs combined may eradicate pests better because of potentiation and synergy (Nicolas et al., 2008 ▶), as well as the lower dose is used. Rotation of acaricides as one of the resistance mitigation strategies is also re-commended when a pest becomes resistant to one class of pesticides, but not to other pesticides with different mode of action (Jonsson et al., 2010 ▶). The histological lesions observed revealed that amitraz could destroy spermatogenic cells and this probably explains the reason rats in group A had reduced ESR and TSR. Since commercial formulations of pesticides incorporate organic solvents such as xylene to the active agent, the toxicity observed in this study could be partly attributed to the organic solvent, xylene and partly to the agent, amitraz.

It was concluded that sub-chronic administration of sub-lethal doses of amitraz could lead to reduced sperm quantity of male animals.

## Conflict of interest

The authors declare no conflict of interest.
